# Morphomics via next-generation electron microscopy

**DOI:** 10.1093/jmcb/mjad081

**Published:** 2023-12-26

**Authors:** Raku Son, Kenji Yamazawa, Akiko Oguchi, Mitsuo Suga, Masaru Tamura, Motoko Yanagita, Yasuhiro Murakawa, Satoshi Kume

**Affiliations:** R IKEN-IFOM Joint Laboratory for Cancer Genomics, RIKEN Center for Integrative Medical Sciences, Yokohama 230-0045, Japan; Department of Nephrology, Graduate School of Medicine, Kyoto University, Kyoto 606-8507, Japan; Advanced Manufacturing Support Team, RIKEN Center for Advanced Photonics, Wako 351-0198, Japan; R IKEN-IFOM Joint Laboratory for Cancer Genomics, RIKEN Center for Integrative Medical Sciences, Yokohama 230-0045, Japan; Department of Nephrology, Graduate School of Medicine, Kyoto University, Kyoto 606-8507, Japan; Multimodal Microstructure Analysis Unit, RIKEN–JEOL Collaboration Center, Kobe 650-0047, Japan; Technology and Development Team for Mouse Phenotype Analysis, RIKEN BioResource Research Center, Tsukuba 305-0074, Japan; Department of Nephrology, Graduate School of Medicine, Kyoto University, Kyoto 606-8507, Japan; Institute for the Advanced Study of Human Biology (ASHBi), Kyoto University, Kyoto 606-8501, Japan; R IKEN-IFOM Joint Laboratory for Cancer Genomics, RIKEN Center for Integrative Medical Sciences, Yokohama 230-0045, Japan; Institute for the Advanced Study of Human Biology (ASHBi), Kyoto University, Kyoto 606-8501, Japan; IFOM—The FIRC Institute of Molecular Oncology, Milan 20139, Italy; Laboratory for Pathophysiological and Health Science, RIKEN Center for Biosystems Dynamics Research, Kobe 650-0047, Japan; Center for Health Science Innovation, Osaka City University, Osaka 530-0011, Japan; Osaka Electro-Communication University, Neyagawa 572-8530, Japan

**Keywords:** comprehensive morphological analysis, next-generation electron microscopy, 3D bioimaging, imaging database, deep learning

## Abstract

The living body is composed of innumerable fine and complex structures. Although these structures have been studied in the past, a vast amount of information pertaining to them still remains unknown. When attempting to observe these ultra-structures, the use of electron microscopy (EM) has become indispensable. However, conventional EM settings are limited to a narrow tissue area, which can bias observations. Recently, new trends in EM research have emerged, enabling coverage of far broader, nano-scale fields of view for two-dimensional wide areas and three-dimensional large volumes. Moreover, cutting-edge bioimage informatics conducted via deep learning has accelerated the quantification of complex morphological bioimages. Taken together, these technological and analytical advances have led to the comprehensive acquisition and quantification of cellular morphology, which now arises as a new omics science termed ‘morphomics’.

## Introduction

It is said that ‘a picture is worth a thousand words’. In line with this sentiment, scientists have been developing tools and techniques to visualize biological specimens for around 400 years. Since Robert Hooke first published ‘Micrographia’ with beautiful illustrations of cells in 1665 (Hooke et al., 1665), the use of light microscopy has led to many important discoveries, not only of various microorganisms but also of cellular components.

**Figure 1:**
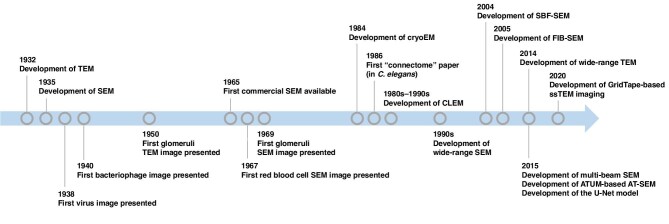
The historical timeline of the EM. Representative milestones in the development of EM are shown.

In 1932, electron microscopy (EM), invented by Von M. Knoll and Ernst Ruska (Knoll and Ruska, 1932), advanced imaging in the field of biology. An exemplary achievement was the microscopic observation of *Escherichia coli* at a magnification of 10000 times (Ackermann, 2011). In the 1940s, EM enabled the discovery of viruses and phage particles, which stimulated the later development of virology (Richert-Pöggeler et al., 2019). Initially, the application of EM in biological research was difficult because of the lack of histological techniques (Marton, 1934); however, with the development of fixing and staining methods using aldehydes and heavy metals, EM was applied more broadly to histology (Knott and Genoud, 2013). The use of EM has also revealed a variety of cellular functions, such as autophagy (Takeshige et al., 1992) and slit structures in kidney glomeruli. In addition, cryo-electron microscopy (cryoEM) modalities have revealed the native structure of cells without the need for chemical fixation (Al-Amoudi et al., 2004). Thus, the technological developments in EM revealed a new world of intracellular nanometre-scale histology. The timeline of key EM observations and technology development is summarized in Figure 1.

In the last decade, two significant trends have emerged in EM research: (i) the broad coverage of two-dimensional (2D) wide-range fields for the simultaneous capture of multiple cells and/or whole tissues at high resolution (Faas et al., 2012); and (ii) advancements in three-dimensional (3D) resolution, offering a volumetric perspective that reveals the morphology of whole cells and the intercellular connections within tissues (Bock et al., 2011; Figure 1). These developments potentially facilitate the handling of large bioimaging datasets and/or the collection of comprehensive morphological data from biological specimens (Wacker et al., 2016). Thus, EM has gained attention as a potential new omics modality. In this review, we discuss the application of ‘big data’ analysis to nano-scale bioimages and highlight the use of deep learning (DL) for state-of-the-art image analysis.

## Implications of EM techniques in biology

EM reveals the structure and localization of cellular components and organelles that can be captured at the nanoscale. This is achieved by using chemically fixed biosamples and a resin-embedded ultra-thin-sectioning EM method (Glauert et al., 1956). To preserve the characteristic microstructures of cell membranes and cellular solutes, specimens must undergo double fixation using a glutaraldehyde agent and osmium tetroxide. Osmium staining is used to selectively visualize intracellular structures as the chemical covalently binds to osmiophilic materials, such as unsaturated fatty acids and biomolecules with unsaturated bonds (Hua et al., 2015), thereby conferring some electron density to the osmiophilic substrates. Additional staining using heavy metals is also performed to improve the contrast of intracellular components. For the preparation of bulk samples larger than a millimetre in size, an *en-bloc* staining method with improved penetration of osmium acid (Mikula et al., 2012) and a prolonged staining technique with sequential osmification have also been developed. Through metal staining, cell morphology can be observed with a clear membrane contrast and the retention of cellular components.

In addition to chemical fixation-based methods, cryoEM modalities have provided a new understanding of biological function and intracellular organization by revealing native cellular structures and molecular details in the absence of usual artifacts caused by dehydration and staining. The native microstructures obtained by cryoEM could be used to verify the cellular images projected by the fixation-based EM method.

Resin-embedding before sectioning is important for EM observation. Epoxy resin is widely used for embedding dehydrated samples. It can permeate into the microstructures of cells and/or tissues, and then the resin-infiltrated sample can be polymerized into a firm plastic that is suitable for ultra-thin sectioning. Fernández-Moránab (1953) proposed a method for producing ultra-thin resin sections using a diamond knife: Trimmed resin-embedded samples were mounted on an ultramicrotome and sectioned at room temperature, usually to <100 nm, using a diamond knife with a wedge angle of 35°–45°. Currently, commercially available diamond knives for ultramicrotomes are around 2–4 mm in width. It is still challenging to grind diamond knives wider than 4 mm using a rake angle sharper than 45°, because the cutting edge must be configured with an acute angle and the wider blade can easily spill during cutting. To maximize efficiency and success during ultramicrotome sectioning, it is necessary to accumulate cutting data as well as consider the basic theory of cutting to determine the quality of ultra-thin sections (Sun et al., 2017).

With the advent of 3D EM methods, the preparation of serial sections from specimen blocks has become possible. Generally, serial sections are prepared using a conventional ultramicrotome by floating the sections on the water surface using a boat attached to a diamond knife (Gay and Anderson, 1954). When several sections have accumulated, they are manually scooped out of the water using a glass or silicon substrate. However, it is currently difficult for even expert technicians to manually prepare and collect several hundred serial sections without missing pieces, and the process has low reproducibility. Furthermore, collecting a large number of sections is time-consuming, and the position of the collected sections varies, which makes it difficult to rearrange the sections. Consequently, new serial-sectioning methods have been proposed. One such method involves the use of an automated tape-collecting ultramicrotome (ATUM), a device that automatically collects serial tissue sections using an ultramicrotome and magnetic polyimide tape (Hayworth et al., 2014). Recently, automated ultramicrotome techniques and diamond knives, particularly for use in continuous floating section preparation, have been developed (Burel et al., 2018). Further details are outlined in the ‘3D resolution bioimaging using EM’ section.

## Transmission EM for wide-area imaging in biology

### Conventional transmission EM and its challenges

In the biological and medical fields, transmission electron microscopy (TEM) is typically used for imaging stained thin sections of plastic-embedded samples (Winey et al., 2014). In TEM, an electron beam with an extremely short wavelength is accelerated to irradiate thin sections (Supplementary Figure S1A). Through the detection of the transmitted and forward-scattered electrons through the thin section, a 2D projected magnified image of the specimen can be obtained at sub-nano-scale resolution. Specimens such as bulk tissues must be sufficiently thin to allow electrons to pass through. The ultra-thin sections are typically ≤100 nm thick because thicker sections cause inelastic electron scattering. These sections are placed on a metal mesh grid for observation.

Some limitations exist when attempting to prepare ultra-thin slices suitable for TEM observation. First, the metal mesh itself interferes with the observation of overlapping tissues. Second, brittle slices are prone to breakage, which restricts imaging time. Third, since large specimens do not fit in a single field of vision (FOV) of the microscope (Preibisch et al., 2009), a controlled system is required to automate the imaging process (Toyooka et al., 2014), and a handling system is needed for large-sized digital images (Kume et al., 2017). These limitations have restricted the use of conventional TEM when observing narrow areas or a relatively small set of cells (Winey et al., 2014). The arbitrary choice of observation areas without proper random sampling or complete serial sectioning of the specimen, which is at present not feasible using TEM, creates bias in TEM observation. Consequently, the current clinical application of EM is limited to assisting in the diagnosis of renal diseases, undifferentiated tumours, metabolic diseases that mainly affect the muscles or nerves, and diseases with unknown aetiologies (Gordon, 2014).

In recent years, progress has been made in overcoming the limitations associated with TEM observations. First, large-sized windows and tough supporting films with uniform thicknesses have been developed to assist in observing wide-range areas (Bock et al., 2011). One such supporting film, the LUXfilm® support film, is a highly transmissive and robust film that is better suited for automatic TEM workflows; however, it produces substantial noise without any noise reduction (Quan et al., 2019). Konyuba et al. (2018) proposed a large-scale silicon nitride window chip deposited using low-pressure chemical vapour deposition as a new support grid for wide-area TEM imaging. This chip is mesh-free, which allows wide-area support for the specimen without creating imaging interference.

A large number of digital TEM images can be captured using an auto-acquisition system with the device (Schorb et al., 2019). When the physical movement of the microscope stage is not sufficiently precise to obtain the required imaging resolution, computational registration and stitching techniques for digital images can be used; these techniques reconstruct single-captured wide-area images from individual tile images. These tiled images are also known as montage images or mosaic images. Toyooka et al. (2014) reported the use of wide-area TEM imaging with a tiled scan of a whole plant cell; this technique successfully produced 3000–5000 digital images with the desired range of observation and comprehensive detection of plant organelles. Faas et al. (2012) performed a large-scale EM analysis known as virtual nanoscopy, a methodology for ultra-structurally mapping regions of cells and tissues as large as 1 mm^2^ at nanometre resolution. Lamers et al. (2020) imaged human severe acute respiratory syndrome coronavirus 2-infected intestinal organoids autonomously using virtual nanoscopy slides and TEM tomography.

**Figure 2:**
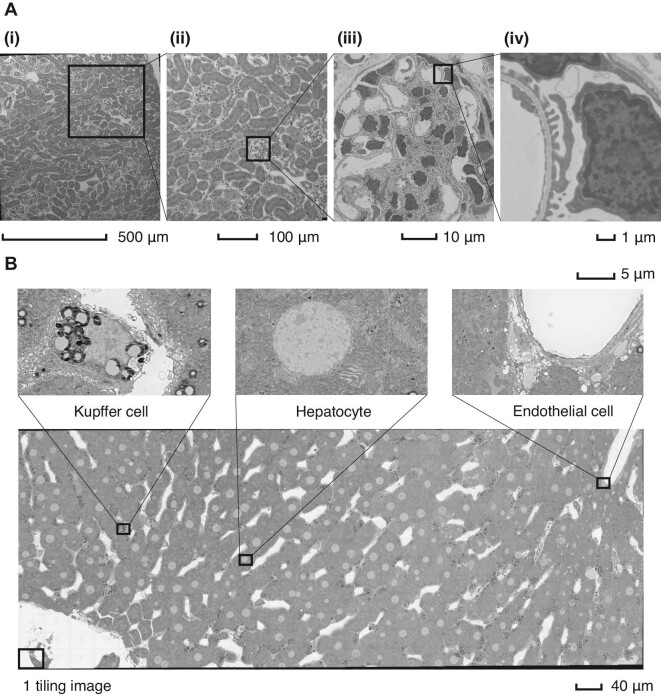
2D wide-area EM imaging. (**A**) Wide-area TEM imaging of a mouse kidney sample. Weakly enlarged images (i and ii) show the simultaneous imaging of multiple glomeruli and renal tubules; strongly magnified images (iii and iv) are conventional EM views containing endothelial basement membranes, podocytes, mesangial cells, and epithelial cells. (**B**) Wide-area SEM imaging of a rat liver section via the backscattered electron detection method. The tiled images provide a view of Kupffer cells, hepatocytes, and endothelial cells in addition to the hepatic lobule with different magnifications.

As shown in Figure 2A, through wide-range TEM imaging, including TEM [JEM-1400/Matataki Flash Camera (2048 × 2048 pixels), JEOL Ltd] with a silicon nitride window chip and an automated montage system, it was possible to obtain a view of mouse glomeruli that consisted of 8500 tiled images. This technique not only preserves the conventional resolution required to capture the basement membrane of the glomeruli and podocyte foot effacement but also enables imaging of multiple glomeruli within the same captured view. Hence, the large 2D EM imaging of a single section described above is a powerful method. However, the morphological information acquired from large 2D areas is still insufficient due to a lack of volumetric viewpoints for tissues and cells. Conventional TEM observation methods still have no way at present to avoid issues such as section thickness effects, over-projection, or missing sections. To this end, new methods for overcoming the incomplete points of large 2D EM imaging are reviewed in the ‘3D resolution bioimaging using EM’ section.

### Scanning-type TEM for large-scale microstructural imaging

The nanotomy project (http://www.nanotomy.org/) and its related works provide systematic virtual nanoscopy studies, mainly using scanning-type TEM in which the electron probe is scanned across the sample and the transmitted signals are detected point-by-point to form an image. This approach enables large-scale morphological imaging of various biosamples, including human pancreas tissue from patients with type 1 diabetes (Boer et al., 2020), human autoimmune blistering disease pemphigus (Sokol et al., 2015), human skeletal muscle biopsies with histological minimal myositis and capillary pathology (Siegert et al., 2021), human haematopoietic stem/progenitor cells (Capala et al., 2015), rat islets of Langerhans (Ravelli et al., 2013), and mouse kidneys and glomeruli (Dittmayer et al., 2018). Recently, Dittmayer et al. (2021) developed a methodology for preparing large-scale digitized samples designed to acquire entire sections free from obscuring flaws using scanning-type EM in transmission mode; this technique will substantially improve the information and throughput gain when analysing experimental and/or clinical samples, including diagnostic muscle, nerve, and kidney samples.

Large-scale EM with energy-dispersive X-ray (EDX) analysis enables the acquisition of elemental composition patterns from the surface of samples and the visualization of traditional grey-scale EM images for composition-based interpretation (Pirozzi et al., 2018). EDX analysis of the rat pancreas has been used to distinguish, for example, cytoplasmic mitochondria and granules via elemental fingerprinting (Scotuzzi et al., 2017). Thus, EDX analysis enables unbiased fingerprinting of cell types, and the functionalities of each cell type can be inferred from elemental fingerprinting.

## Large-scale bioimaging using scanning EM

### Application of scanning EM in biology and recent progress

Scanning EM (SEM) involves the use of a different type of electron microscope from that used for TEM (Supplementary Figure S1B). The development of SEM began in the 1930s, and the first applications of SEM in materials science were reported in 1955 (Smith and Oatley, 1955). SEM was first commercialized in 1965 (McMullan, 2008), ∼30 years after TEM. In SEM, the incident electron probe scans across the surface of a specimen in a raster fashion, and the interaction between the relatively heavy elements containing the sample and the impacted electrons produces various types of emissions, including secondary electrons, backscattered electrons, and characteristic X-rays (Suga et al., 2014). By detecting such emission types, SEM creates images that reflect the topological contrast or compositional information of specimens as signal intensities in digital images (Suga et al., 2014). Typical SEM measurements do not require the transmission of electrons through the sample. Therefore, SEM can be used for surface observation of semi-thin sections and bulk specimens such as the surface of kidney glomeruli (Conti et al., 2018) and blood cells (Hartigan et al., 2016). However, traditional SEM images lack the characterization of internal ultra-structures due to the relatively lower signal under high-magnification imaging conditions. Thus, the application of SEM in diagnostic pathology is limited (Cohen Hyams et al., 2020).

Fortunately, recent progress in SEM imaging has led to new SEM utilities, e.g. scanning across ultra-thin sections of resin-embedded specimens under conductive support. Field-emission SEM produces high-resolution images because of the smaller spot size from the emitters, the negative stage bias potential, and the improved sensitivity of multiple electron detector systems (Suga et al., 2014), even when the ultra-thin tissue sections are <100 nm. The backscattered electron detection by SEM when using resin-embedded sections provides a reverse contrast of the view that is conventionally possible when using TEM. Although the contrast of the backscattered images is reversed, the quality of the images is sufficient to enable general morphological analysis from TEM observations. Observation of tissue sections through a combination of SEM and resin-embedded sectioning is also known as the section SEM method.

For the SEM observation, various shapes of sample stands can be used provided that they are not made of electrically charged materials. A silicon wafer is a typical base used for biological SEM specimen observation; indeed, huge specimen bases, e.g. 10 cm-diameter wafers, are available. Silicon wafers also adhere well to ultra-thin sections. Sections scooped up on the wafer can be stably stored, even when the section is large. Because SEM is typically used for surface observation of bulk samples, the sample storage space is designed to have a large XYZ dynamic range. In other words, the stable fixation of a sample on a sample board has made it possible to observe samples over a long period of time. The large XY dynamic range facilitates the introduction of relatively large sections (i.e. several millimetres), multiple sample sets, and hundreds of sections into the instrument at the same time. The use of the range in the Z-direction has made it possible to include microfabrication methods, such as knife cutting and laser cutting, in the sample chamber.

### Wide-area imaging using SEM

To practically produce a fish-eye perspective view, early panoramic imaging with SEM was performed in the 1990s (Oho et al., 2000). To convert mosaic images obtained by SEM into a combined image, image stitching algorithms have also been developed. For wide-range SEM imaging, Brantner et al. (2016) demonstrated large-area and high-resolution mosaic imaging of a 2.5 mm × 1.8 mm mouse spinal cord resin section (at a biologically relevant scale) using the workflow of Chipscanner's laser interferometer stage, FOV mapping, and an image stitching technique. Kataoka et al. (2019) indicated that stitching SEM enabled the observation of an entire pulmonary alveolus with influenza virus particles in a resin section. More et al. (2011) applied a montage SEM imaging technique to quantify the number, myelination, and size of axons in the rat fascicle using a computer-assisted axon identification and analysis method. Kume et al. (2017) reported an imaging database of wide-range montage SEM images and their metadata for various tissues, including those from the kidney, liver, and brain cortex regions of rodents and cultured human cells. Figure 2B shows imaging data obtained using wide-area montaged SEM images of a rat liver. We integrated more than 1110 images to reconstruct the rat liver leaflet in this large-area image (1 mm × 0.6 mm). Strikingly, we were able to observe the whole liver lobule while preserving the spatial resolution. The use of wide-area EM imaging avoids the arbitrary selection of target regions in experimental or diagnostic specimens and enables the efficient and comprehensive observation of biological tissues without bias.

### Multi-beam EM

SEM imaging can be more time-consuming than TEM imaging due to raster fashion scanning. To speed up SEM imaging, several methods have been developed: (i) capturing images with a higher-speed single-beam; (ii) imaging different sections in parallel on multiple EM devices; and (iii) parallelized imaging of the same section using multiple scanning beams.

As a method of parallelized imaging, Eberle et al. (2015) demonstrated a throughput imaging technique with multi-beam SEM. In this system, 61 electron beams are scanned over the sample with one global scanner, and secondary electron signals are detected for each scan position of each beam. Multi-beam SEM simultaneously produces 61 montaged SEM images as a hexagonal FOV. In the resultant images, all the membranes of the neural tissue were clearly visible, and the intracellular organelles were distinguishable (Eberle et al., 2015). Pereira et al. (2016) reported that a surface area of 5.7 mm^2^ could be imaged in a human femoral neck tissue sample, resulting in 897 hexagonally shaped multi-beam FOVs comprising ∼55000 high-resolution image tiles and 75000 megapixels. Thus, multi-beam EM systems contribute to the high-speed collection of digital images. Recently, Delmic developed FAST-EM, which is categorised as a scanning-type TEM system (Fermie et al., 2021). FAST-EM uses a 64-multi-beam system combined with the detection of transmitted electrons. With a significant improvement in imaging speed, the FAST-EM technique could replace conventional single-beam EM in the future.

## 3D resolution bioimaging using EM

### Implications of 3D resolution for ultra-microstructural observations

**Figure 3:**
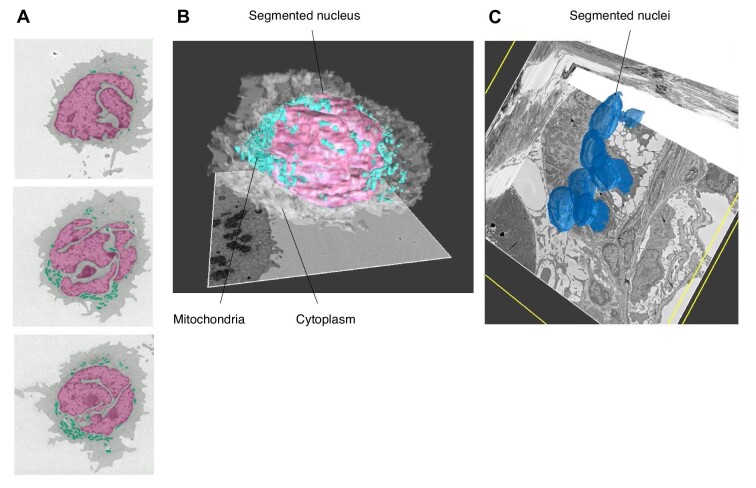
3D volume EM imaging using AT and SEM. (**A**) Serial sections of an NB4 cell (an M3 acute myeloid leukaemia cell). The shape of the nucleus is highly variable even within a single cell. (**B**) 3D reconstruction of the NB4 cell using about 130 serial sections shown in **A**. The nuclei, cell body, and mitochondria (high electron density organelles) of the cell were segmented. (**C**) 3D reconstruction of the macula densa in the distal tubules of a mouse kidney glomerulus. The nuclei of the macula densa were segmented.

To obtain histological and cellular images of targeted 2D regions, the use of ultra-thin section EM techniques with resin-embedded samples is widely accepted and has led to new biomedical discoveries (Knott and Genoud, 2013). Indeed, a cellular image obtained from only one tissue section contains substantial biological and medical information. However, the thickness of ultra-thin sections is 50–200 nm. Assuming that the actual size of a cell is ∼10 μm, one ultra-thin section of approximately one-fiftieth to one-two hundredth of the total cell volume cannot be used to entirely interpret cellular events (e.g. the steric and complex cellular communications in living tissues) and the appearance of characteristic cells and compositions in diseases (Boer et al., 2020). There is a very plausible relationship between ultra-thin sections and cell volume. In most cases, even within the same cell, the shape of the cell nucleus differs greatly depending on the cutting angle and position of the cross-section (Figure 3A). In other words, when a cell image is observed in a cross-section, it is difficult to precisely describe the whole-cell morphology. In such cases, visualizing the entire morphology of target cells or tissue regions at 3D resolution is required.

To realize 3D-directed resolution in EM techniques, observing multiple tomographic images for each cross-section one-by-one is a reasonable method (Barajas, 1970). Thus, the generalization of stereoscopic EM techniques will lead to a deeper understanding that would otherwise not be obtained using conventional 2D EM techniques. Briefly, volumetric EM images can be obtained by either SEM or TEM imaging (Supplementary Figure S2). We reviewed four representative volumetric EM techniques in the following sections.

### Focused ion beam SEM

Focused ion beam SEM (FIB-SEM) is used to observe the surface of a specimen milled by an ion beam on the sample stage of the microscope (Drobne et al., 2005). By repeatedly and alternatively exposing and imaging the new top surface, serial images are captured, although the cutting surfaces cannot be preserved (Supplementary Figure S2A). FIB-SEM offers the best Z-axis resolution at 4–5 nm; thus, it is suitable for mesoscale observations, such as the observation of cellular organelles.

As the area of observation is enlarged via FIB-SEM, this technique is commonly applied to various biological samples. Moreover, the outstanding Z-axis resolution of FIB-SEM is suitable for observing intracellular events and organelles (Weigel et al., 2021). Using FIB-SEM, Miyazono et al. (2018) demonstrated dramatic mitochondrial structural changes that were triggered by the loss of mitochondrial membrane potential. Notably, Xu et al. (2020) enhanced the FIB-SEM system by accelerating image acquisition; the speeded-up system allowed imaging of a *Drosophila* brain at 106 μm^3^, which serves as a powerful dataset in brain connectomes. Furthermore, Xu et al. (2021) reported volumetric imaging datasets of whole-cell architecture with the finest possible isotropic resolution (about 4 nm square voxels) using FIB-SEM, provided as open access data via OpenOrganelle (https://openorganelle.janelia.org/) (Weigel et al., 2021), which allows the comprehensive study of cellular organelle morphologies (Müller et al., 2020).

### Serial block-face SEM

Serial block-face SEM (SBF-SEM) is used to observe an exposed sample surface cut using a built-in diamond knife (Supplementary Figure S2B). Compared with FIB-SEM, SBF-SEM facilitates the handling of a broader area as well as faster sample sectioning, but the XY resolution and contrast of images are limited. SBF-SEM not only showed the power to reconstruct 3D tissue nanostructures but also enabled the visualization of neural circuit reconstructions in neuroscience research (Denk and Horstmann, 2004). The largest mammalian cerebral cortex dataset yielded a reconstruction ∼300-fold larger than that in previous reports, which allowed the analysis of axonal patterns (Motta et al., 2019). Other reports have also shown the feasibility of SBF-SEM in 3D EM studies, including studies of *Drosophila* epithelium (Daniel et al., 2018), rat podocytes (Ichimura et al., 2017), and the whole structure of cultured cells (Spiers et al., 2021).

### Array tomography

Array tomography (AT) is also used to achieve stereoscopic EM (Supplementary Figure S2C). In the AT method, serial ultra-thin sections are prepared from a resin-embedded block using an ultramicrotome, and then the same site in each section is observed sequentially using TEM or (primarily) SEM. Unlike other methods, the AT method is notable for its capacity to preserve thin sections, which could then be re-observed later. The resolution of the Z-axis in the AT method is the thickness of the section, which is ∼50–100 nm.

Notable results of combined AT and TEM include whole-brain imaging of adult *Drosophila* using a custom high-throughput serial-section TEM (ssTEM) platform developed by Zheng et al. (2018). This volumetric morphology obtained by ssTEM has contributed to the mapping of brain-spanning circuits and accelerated neuroscience research. In parallel with the development of ssTEM, AT combined with SEM was proposed in 2007 (Micheva and Smith, 2007). This combination method has been used to study various biological samples (Wacker et al., 2015). In addition, researchers could successfully generate a 3D volume EM image of a human leukaemia cell and the macula densa in the distal tubules of a mouse kidney glomerulus using AT combined with SEM (Figure 3B and C).

Combining the AT method with SEM potentially allows wide-area volumetric observations (Wacker et al., 2016). This technique is also known as the serial-section SEM method. The SEM-based serial-sectioning method is suitable for relatively large samples because it collects larger-area serial thin sections onto the sample substrate. However, the AT method is generally challenging because it is difficult to manually prepare continuous ultra-thin sections of hundreds to thousands of samples. To improve the AT technique, customized AT methods, such as magnetic collection of ultra-thin sections (Templier, 2019), use of a modified AT-boat diamond knife (Burel et al., 2018), and tape collection of sections (Kasthuri et al., 2015), have been developed. Among these methods, the use of an ATUM tape collection system has facilitated the automatic collection of tissue serial sections and volumetric SEM. This ATUM-based AT-SEM method was used to clarify the sub-volume of the mouse neocortex from ∼2000 serial sections (Kasthuri et al., 2015) as well as all myelinated axons of the zebrafish brain from 16000 serial sections (Hildebrand et al., 2017). Witvliet et al. (2021) used the ATUM-SEM system to reconstruct the full brains of eight isogenic *Caenorhabditis elegans* individuals across postnatal stages in an age-dependent manner, which provided insights into the mechanism of connectome development during brain maturation. These obtained datasets are provided in the Brain Observatory Storage Service & Database (https://bossdb.org/; Vogelstein et al., 2018). The ATUM devices are mainly used in connectome research, which aims to elucidate the network of neurons in the entire brain (Kasthuri et al., 2015), but they may be used in other research fields. Moreover, the use of such large-scale stereoscopic EM techniques to analyse the microstructures of pathological conditions is expected to improve our understanding of disease-specific structures that could not be obtained using conventional EM techniques.

### 3D imaging using high-speed TEM

To develop high-throughput TEM imaging methods, Graham et al. (2019) used a tape-based, reel-to-reel pipeline that combines automated serial sectioning and a TEM-compatible tape substrate, GridTape. This acquisition platform provides nanometre resolution imaging at fast rates via TEM. Based on this pipeline, multiple-scope parallel imaging using a 50-MP camera has enabled image acquisition of a >1 mm^3^ volume of the mouse neocortex, spanning four different visual areas at synaptic resolution, in less than 6 months; in turn, this approach has yielded a >2 petabyte dataset from over 26500 ultra-thin tissue sections (Yin et al., 2020). In addition, Phelps et al. (2021) applied GridTape-based ssTEM imaging to acquire a synapse-resolution dataset containing the ventral nerve cord of an adult female *Drosophila*. The complete connectivity maps provided a deeper understanding of the control of the nervous system (Phelps et al., 2021). Since TEM offers much faster imaging than SEM, this research could be applied in areas that require further broad observation with precise imaging.

### Comparison of 2D and 3D EM imaging

In general, 3D EM imaging carries a much higher spatial resolution than 2D EM. However, the current 3D application needs to be considered based on the target for observation. For example, while the fixation methods used in 3D EM are the same as those used in 2D EM, some of the 3D SEM methods, such as FIB-SEM and SBF-SEM, need *en-bloc* staining (Mikula et al., 2012) to ensure that the whole sample block is appropriately stained by heavy metals. Also, different techniques and modalities for 3D EM imaging can be chosen based on the imaging area (X–Y-axis), X–Y-resolutions, Z-axis resolution, imaging speed, sample reusability (Hua et al., 2015), and re-observation, as mentioned above. For example, while FIB-SEM offers the best Z-axis resolution (4 nm), it has limited capturing areas. The AT method covers broader capturing areas, but is still technically challenging to produce large datasets of complete sections in a high-speed manner. Currently, the best imaging method should be considered based on the sample characteristics and research objectives.

## Correlative EM and multimodal imaging

### Multimodal imaging and its challenges

To ensure accurate morphomics, it is imperative to minimize sampling and experimental biases. This can be achieved by employing a low-resolution screening approach across entire tissues to identify the 3D structures of interest and utilizing a targeting approach to assess the localizations of specific structures and cellular markers. Indeed, a correlative microscopy approach using multimodal imaging modalities combined with EM is a key strategy to achieve this. In the following sections, we review the screening and targeting approaches of multimodal imaging with light microscopy and X-ray computed tomography (X-ray CT).

### Correlative light microscopy and EM

The body can be understood in more detail if tissue morphological functions and macromolecular fingerprinting of entire tissues can be estimated at the nano-level, which could be difficult to achieve using only EM-based morphomics. For example, distinguishing between excitatory and inhibitory neurons cannot be achieved based solely on morphology (Collman et al., 2015). To overcome this problem, correlative EM, combining EM and other imaging tools, could be used to better understand molecular functions and other factors. Correlative EM is also useful for screening or targeting specific structures, especially cellular markers. A well-established example is the combination of light microscopy and EM, known as correlative light and electron microscopy (CLEM) (Boer et al., 2015). The concept of CLEM was first proposed in the 1980s. First, samples are imaged using a light microscope to detect entire histological morphologies or fluorescence signals, after which the samples are subjected to nano-resolution EM imaging. Correlation imaging could be achieved either by sharing the same FOV for both modalities or by transferring samples in tandem.

To screen for structures of interest, Ronchi et al. (2021) developed a volume EM workflow that combines fluorescent labelling and FIB-SEM, thereby enabling correlative targeted imaging of animal mammary gland organoids, tracheal terminal cells, and ovarian follicular cells, providing a framework for volumetric EM analysis of specific single cells within large tissue samples. CLEM has also been applied to studies of the mouse brain (Fang et al., 2018), whole model organisms (Burel et al., 2018), and various tissues.

When targeting specific structures, Trzaskoma et al. (2020) applied CLEM to reveal 3D chromatin folding by combining DNA fluorescent *in situ* hybridization with SBF-SEM. Oorschot et al. (2021) published a workflow integrating the Tokuyasu technique to preserve protein antigenicity and investigated neural stem and progenitor cell populations. In addition, 3D CLEM combined with the CryoChem technique allows for high-quality ultra-structural preservation, making it broadly applicable to cultured cells and tissue samples (Tsang et al., 2018). The CLEM method can also be used to target specific transgenic proteins via the engineered peroxidase gene *APEX2* (Lam et al., 2015) as a labelling probe for both EM and light microscopy. This system has been successfully implemented to track lysosomes in dendrites (Goo et al., 2017), visualize the localization of endoplasmic reticulum chaperonin (Mavlyutov et al., 2017), and visualize the outer endoplasmic reticulum and mitochondrial membrane (Hung et al., 2017). Recently, the highly sensitive APEX-Gold method was used for genetic tagging in 3D EM.

For correlation of live-cell imaging, Fermie et al. (2018) analysed the dynamics of individual GFP-positive structures in HeLa cells and then correlated these images with those obtained from FIB-SEM. This approach overcame the limitation of EM in visualizing live cells. Betzig et al. (2006) first introduced a combination of super-resolution light microscopy and EM (super-resolution CLEM) to image specific target proteins in thin sections of lysosomes and mitochondria. Currently, super-resolution CLEM can achieve a resolution of 20–50 nm, although sample distortion becomes a problem at <10 nm resolutions. This technique has also been used to visualize the Golgi apparatus (Kurokawa et al., 2019), mitochondria, and other organelles (Hoffman et al., 2020).

### Correlative X-ray CT and EM

X-ray CT has been applied to biological tissues or cells to obtain 3D morphology data at almost the single-cell level (Sakdinawat and Attwood, 2010). In practical terms, observing ∼10-μm cells requires sub-micro-resolution potential in the CT device. When observing intracellular structures, synchrotron radiation X-ray should be used. Currently, utilizing a CT device for single-cell imaging at the intracellular level is considered a special case. However, in this section, we explore cellular tissue analysis, encompassing single-cell imaging with CT devices, and extend the discussion to the correlation between CT and EM (correlative CT and EM).

To date, a few examples of X-ray CT have been applied in biological research. In the early 1980s, the micro-CT technique was developed to achieve 3D observations at micrometre resolutions. This technique can be used to obtain a projection image of a sample by X-ray irradiation at wavelengths ranging from ∼1 pm to 10 nm. Compared with the 1–2 mm resolution in conventional medical CT scans, micro-CT tomography results in a higher spatial resolution of 1–50 μm (generally approximately a resolution of 5 μm per voxel) (Merkle and Gelb, 2013). As spatial resolution depends on the focal spot size of the X-ray source, relatively small sample pieces (<10 mm in size) could be investigated at micrometre-level resolution using micro-CT. Moreover, micro-CT imaging provides high-contrast results, especially for tissues with high or low X-ray permeability (e.g. lungs and bones, respectively), without the need for special sample preparation. However, particularly in soft tissues, including the brain and renal cortex, suitable staining techniques are required to increase the absorption-based contrast of tissue structures (Metscher, 2009). Micro-CT has been applied to visualize stained specimens, including rodent kidney nephrons (Busse et al., 2018), mouse liver structures (Müller et al., 2018), and mouse embryos (Tamura et al., 2013; Dickinson et al., 2016). In addition, the phase-contrast X-ray CT approach can generally be applied to unstained specimens (Töpperwien et al., 2018). Overall, this modality has become a promising method in morphomics.

For 3D non-destructive targeting of a region of interest in a specimen, EM analysis correlated with the X-ray CT modality has been proposed. In particular, correlative micro-CT and EM have been applied to clarify neural 3D structures in mouse brain tissues (Bushong et al., 2015). Using silver impregnation staining applied to neurons is also feasible in correlative workflows. Karreman et al. (2016) demonstrated the *in vivo* tracking of single tumour cells using multimodal imaging, including X-ray CT and EM, which is expected to be broadly applied in various biological fields.

Certain CT devices even offer sub-micro-resolution (Müller et al., 2017), e.g. nano-CT, which could be used as a synchrotron radiation-based CT setting and soft X-ray with relatively low penetrating power. This resolution could enable cell-level observations (Müller et al., 2018) that could further facilitate correlative analysis combined with EM. Interestingly, Kuan et al. (2020) demonstrated that X-ray holographic nano-tomography could be used to image large-scale volumes with sub-100-nm resolution in *Drosophila melanogaster* and mouse nervous tissue, thereby enabling close reproduction of EM images. Moreover, the multiple scanning approaches allow for comprehensive cataloguing mechanosensory neurons and tracing individual motor axons from muscles to the central nervous system (Kuan et al., 2020). Nano-scale X-ray CT could then bridge a key gap that helps move towards EM resolutions. Furthermore, the integration of nano-scale CT and EM has been used to study drug resistance-related mitochondrial morphology in human colon carcinoma cells (Moscheni et al., 2019). A parallel-beam CT method can achieve significantly faster image acquisition and comparable or even better resolution. Overall, the X-ray CT modality displays the potential to be used not only for regional targeting prior to correlative EM analysis but also for morphomics analysis of parenchymal morphology at nano-scale resolution.

## Large bioimaging datasets and comprehensive bioimage analysis

### The morphome as another layer of omics data

**Figure 4:**
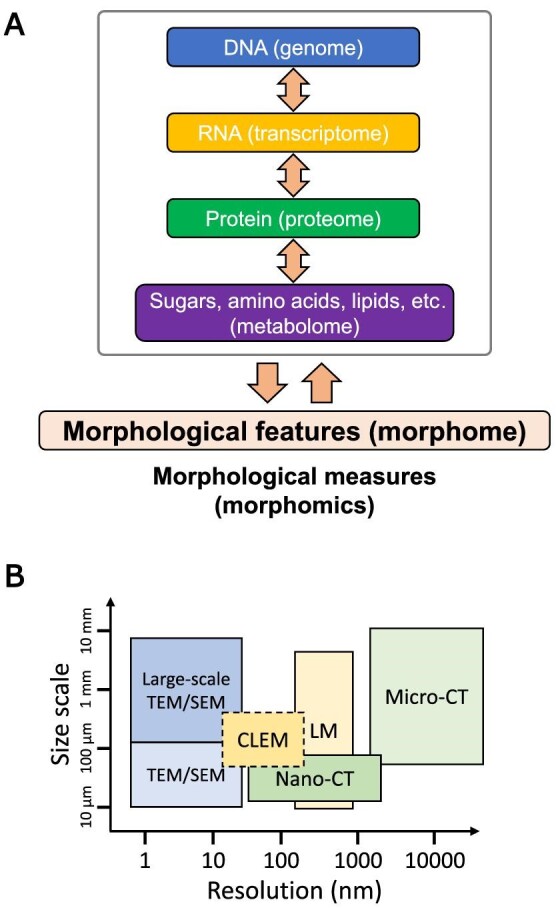
Overview of morphomics analysis of biological tissue using various imaging approaches. (**A**) The morphome refers to the totality of the morphological features of cells or tissues in an organism. It is the result of molecular dynamics, including its DNA (genome), RNA (transcriptome), protein (proteome), and metabolic (metabolome) information. (**B**) Morphologically, various types of tissues, cellular structures, and organelles in the living body range from centimetre to nanometre scales, which can be observed via different imaging techniques. In certain cases, the continuous nature of the morphome cannot be quantified by one microscopy technique alone. To compensate for the weakness of each microscope, a combination of imaging-based methods, known as correlative microscopy mentioned previously, is required to observe the biological nature and process massive amounts of multi-layered morphome data. LM, light microscopy.

The morphome refers to the totality of the morphological features of cells or tissues in an organism (Mayhew, 2015). It is the result of molecular dynamics (Lucocq et al., 2015), including its DNA (genome), RNA (transcriptome), protein (proteome), and metabolic (metabolome) information (Figure 4A). Most morphological data are imaging data, which at first glance differ from the sequencing data that are mainly used as omics data in molecular biology (Kume and Murakawa, 2020; Kume, 2021). Morphologically, the sizes of various types of tissues, cellular structures, and organelles in the living body range from centimetre to nanometre scales (Figure 4B), which can be observed and quantified via different imaging techniques. In certain cases, the continuous nature of the morphome cannot be quantified by one microscopy technique alone. To compensate for the weakness of each microscope, a combination of imaging-based methods, known as correlative microscopy mentioned previously, is required to observe the biological nature and process massive amounts of multi-layered morphome data (Figure 4B).

As EM device technologies have advanced, the acquisition speed of EM imaging has dramatically accelerated, and EM-based imaging techniques have been leveraged to study the complexity of organisms, offering high-resolution insights in both 2D and 3D dimensions. Indeed, imaging data can be produced at a level comparable to the vast amount of genomic data generated by next-generation sequencing. For example, wide-area imaging produced using single-beam SEM can acquire several tens of gigabytes of data in a single day of imaging (Kume et al., 2017), whereas 2D/3D imaging produced using multi-beam SEM can acquire hundreds of gigabytes or a terabyte scale of image data (Eberle and Zeidler, 2018). Moreover, the latest EM methods, such as high-speed ssTEM methods (Yin et al., 2020) and ATUM-based AT and multi-beam SEM methods (Shapson-Coe et al., 2021), can generate petabyte-scale image data.

These levels of imaging data can be used to systemically measure and quantify large morphological fingerprints and diverse biological phenomena. Further quantitative morphology analysis could be applied to study biological functions. Such comprehensive approaches have led to the treatment of the resultant large imaging datasets as new omics information, which is termed morphomics (Figure 4A). This involves the integration of comprehensive (big) morphology data and bioimaging informatics, which will result in the discovery of unknown features, but there is still a bottleneck in imaging data mining.

### Towards the handling of massive bioimaging datasets

Just over 20 years ago, film photographs were still in mainstream use for EM (Heuser, 2000), whereas nowadays film-based images have been converted to high-resolution digital images. During this period of development, advances in infrastructural technologies, such as digital image-archiving, faster network communication, improved computing performance, and increased storage disk capacity, have facilitated the acquisition of large-scale digital bioimage data and enabled the practical handling and processing of images (Eliceiri et al., 2012). It is now possible to operate with hundreds of gigabytes or terabytes of images even in a laboratory setting. Efforts are being made in the field of bioimaging data operations to handle such huge imaging datasets for data storage, sharing, and reuse in a standardized manner (Swedlow et al., 2021).

Open-source data and data accessibility are critical to the sharing of bioimaging data (Swedlow et al., 2009). However, it is necessary to construct a descriptive format and data repository, so-called metadata and an image database, respectively, prior to distributing bioimaging data (Ellenberg et al., 2018). The Open Microscopy Environment (OME) consortium works to produce imaging metadata and public image archives in the medical and life sciences (Swedlow, 2007). The OME is an open-source software framework developed to address standards for sharing multidimensional and heterogeneous image data and analysis results mainly from light optical microscopy (Goldberg et al., 2005). However, standardized metadata that describe EM experiments, including bioresources, measurement conditions, and image formats, have yet to be developed; thus, integrated analysis of imaging data with other metadata has remained difficult (Kume et al., 2021). Therefore, we previously proposed the development of Resource Description Framework (RDF)-based microscopy metadata to describe EM experiments and their imaging datasets based on the data model of OME metadata (Kobayashi et al., 2018), and this initial work on the RDF-based representation of OME metadata was completed in collaboration with the OME community (Hammer et al., 2021). We also offered a combination of an ontology-based imaging metadatabase for EM images and an image viewer, which were distributed in a machine-readable web form (Kume et al., 2017). Taken together, the activities of the OME community and related works accelerate the sharing and reuse of microscopy images, including EM images, through their proposed metadata and various frameworks for biological researchers.

The field of microscopy technology is rapidly evolving, leading to increasingly large and complex bioimaging data. At present, metadata arrangements for bioimaging, including EM, have been discussed internationally towards the reuse of microscopy data (Sarkans et al., 2021). For unlocking the potential of bioimaging data, Recommended Metadata for Biological Images (REMBI) was proposed to be a systematic archive of publicly available light microscopy and EM data and metadata (Sarkans et al., 2021). The categories of metadata covered by REMBI were converged to be the top-level metadata elements, such as the study component, biosample, specimen, image acquisition, image data, image correlation, and analysed data, which integrate bioimaging data from different experiments and modalities. Through community-driven activity, the barriers to data sharing and reuse of EM images should be reduced in the future.

As a prototype of the first open online repository to link imaging and molecular data, Williams et al. (2017) launched the Image Data Repository (IDR; https://idr.openmicroscopy.org/) in 2017. This platform stores curated bioimaging data of cells and biological tissues from several imaging modalities, including multidimensional microscopy and digital pathology while integrating image data and metadata and phenotypic information from several studies into a single resource. Currently, the IDR platform distributes some EM datasets, including images of chromatin organization (Miron et al., 2020) and intestinal organoid (Lamers et al., 2020). The IDR used the Bio-Formats software library to provide semantic elements that describe the imaging metadata as specified in the OME Data Model (Williams et al., 2017). This format is a general description of microscopic attributes and is not specific to detailed EM descriptions.

The CELL Image Library (CIL; http://www.cellimagelibrary.org) is a searchable database that archives several thousand community-submitted cellular images, some of which are linked to publications and ontologies as well as basic descriptions and technical details (Orloff et al., 2013). The CIL uses the OME-XML data model (Goldberg et al., 2005) and displays cell type, cellular component, image type, image mode, and biological context while using ontologies as a descriptive vocabulary.

The Electron Microscopy Public Image Archive (EMPIAR; https://www.ebi.ac.uk/empiar/) (Iudin et al., 2016) shares cryoEM data. Most EMPIAR datasets include particle images and 3D tomograms of macromolecules obtained using cryoEM, but they currently contain more specialized image resources, such as volume EM images of epoxy-embedded tissue and cell samples and X-ray microscopy images. The image sets include basic imaging conditions, such as image category, image format, number of images or tilts, frames per image, image size, pixel type, and pixel spacing. At present, discussions are ongoing within the EM community about establishing a routine for depositing volume EM data along with their metadata into EMPIAR. Actually, the EMPIAR datasets contain several 3D EM datasets of epoxy-embedded tissue and cell samples. For example, a 3D imaging dataset of the HeLa cell line obtained using SBF-SEM (EMPIAR-10094; Spiers et al., 2021) consists of 518 cross-sectional images with a size of 8192 × 8192 pixels; the dataset is nearly 130 gigabytes in size, indicating that the EMPIAR covers a wide range of biological samples. Taken together, the EMPIAR data model was still expanded by the relevant communities to include essential information about the EM experiment.

To date, the development of these public bioimage resources is at an early stage. Further accumulation of imaging data and the development of integrated platforms are highly desirable. Recently, public image databases such as IDR (Williams et al., 2017) and nanotomy (Ravelli et al., 2013) have more commonly provided large-scale, zoomable datasets via zoomable maps, and they can be accessed in a zoomable fashion on their database system. Representative lists of zoomable public datasets are summarized in Supplementary Table S1. It is expected that increasing the availability of imaging datasets will further stimulate imaging research and the development of novel imaging technology.

### Bioimage analysis using DL

Conventionally, every image was examined manually. Little progress has been made in the methods used to analyse EM bioimages over time. EM bioimages possess black-and-white contrast and a variety of morphological features. When using classical image analysis methods, it has been difficult to recognize and decode EM bioimages. For example, in semantic segmentation, which is used to extract a particular region in an image, the use of classical deductive methods has failed to identify a mathematical solution for the morphological features of a particular region among various other morphologies. In most EM research, comprehensive quantification, including automatic segmentation, could not be achieved even after large-scale imaging datasets are acquired. In an attempt to resolve these issues, the current best practice is to apply cutting-edge informatics or artificial intelligence (AI) approaches (Min et al., 2016) to quantify the microstructures in EM bioimages.

Image analysis via AI techniques, such as machine learning (ML) and DL, has received substantial attention in the fields of biomedicine and imaging research (Laak et al., 2021). Inductive analysis using supervised data has been used to identify characteristic changes in morphology. Initially, such AI techniques were applied in neuroanatomy research. Kaynig et al. (2010) demonstrated a fully automated 3D segmentation process of thin, elongated cell membrane structures of dendrites across 30 sections in TEM images. They achieved this by extracting features from the images and subsequently constructing a classifier using a Random Forest, an ML method based on ensemble learning. Turaga et al. (2010) presented an affinity graph (the X, Y, and Z-direction information) computation that used a four-layered convolutional neural network (CNN) trained with the supervised dataset, resulting in 3D reconstructions of neurites with ∼90% segmentation accuracy in a 3D EM dataset of rabbit retina tissue. These were the original applications of ML and DL in connectomics studies. Subsequently, AI technologies have become indispensable for bioimage analysis. Currently, DL models, which use an exquisite combination of multi-layered CNNs for learning, are the state-of-the-art technology for image recognition; they can extract morphological features, such as the cell body and nucleus, from cellular images via complex networks (Litjens et al., 2017).

For the quantification of bioimaging data by DL, the U-Net model was proposed by Ronneberger et al. (2015). The model has encoder and decoder parts with multi-layered CNNs and contracting paths between the encoder and decoder. U-Net targets segmentation tasks in a small number of imaging datasets with large feature types that are unique to the bioimaging dataset. The U-Net model substantially improves performance in segmentation tasks such as cell division tracking and neuronal cell membrane segmentation (Falk et al., 2019). In addition, 3D applicable models have been developed as an extension of 3D volume data. Therefore, the U-Net method is a leading technique that serves as a foundation for biological image analysis.

On the other hand, some approaches for semi-automatic segmentation have been proposed to suppress the computation cost and the accumulation of the training dataset. Semi-automatic segmentation refers to the technique whereby the initial automatic segmentation is followed by manual checking and editing of the segment boundaries for the next calculation of automatic segmentation step-by-step. Among the semi-automatic segmentation platforms, the Volume Annotation and Segmentation Tool provides a simple user interface for real-time manual and semi-automatic labelling for the exploration and analysis of large 3D imaging datasets (Berger et al., 2018). Additionally, UNI-EM was developed as a unified environment for CNN-based segmentation of EM images (Urakubo et al., 2019), including the procedures of ground truth generation, training, inference, postprocessing, proofreading, and visualization. The UNI-EM incorporates a set of 2D CNNs, i.e. U-Net, ResNet, HighwayNet, and DenseNet, and a 3D CNN-based segmentation algorithm. Thus, these platforms would allow precision and efficiency in quantitative estimates of the comprehensive morphome.

To use ML and DL techniques universally, the CDeep3M tool can perform image segmentation in cloud computing (Haberl et al., 2018). A generalist DL model for cellular segmentation, the so-called Cellpose model, was proposed that can be precisely applied to the segmentation of cells from a wide range of image types (Stringer et al., 2021). Datasets for the performance estimation of DL models were proposed, including the NucMM dataset for 3D Neuronal Nuclei Instance Segmentation (Lin et al., 2021) and the MitoEM dataset for 3D Mitochondria Instance Segmentation (Wei et al., 2020). Recently, the DL model has also been ported to R, and typical models for segmentation are available in the ANTsX ecosystem (Tustison et al., 2021). In addition, we have begun distributing supervised bioimaging datasets in the R array format that can be used in the analytical workflow in the R environment as the BioImageDbs project via the Bioconductor ExperimentHub platform (https://doi.org/10.18129/B9.bioc.BioImageDbs).

In contrast to these successes in bioimage data analysis, automated segmentation tasks related to EM images remain challenging. A benchmark report comparing seven published models for EM images showed that their performances were still highly variable (Khadangi et al., 2021). In recent years, however, successful cases of EM image analysis using DL have been reported in neuroanatomy research and in other fields. In neuroanatomy research, Lee et al. (2017) reported a residual symmetric U-Net architecture that achieved an approximately 2%–3% error rate for an EM imaging dataset of mouse neurites (Kasthuri et al., 2015) in the SNEMI3D challenge; thus, the system surpassed the human accuracy value provided at that time. Januszewski et al. (2018) used a flood-filling network to trace neurons in a dataset from a zebra finch brain obtained using SBF-SEM; they achieved high-precision automated reconstruction of neurons with a mean error-free neurite path length of 1.1 mm. For multiple segmentation tasks with EM images, the transfer of learning from pre-trained models using the CEM500K dataset was effective for the transferability of learned features, indicating that a large amount of training data is important for encoding bioimages (Conrad and Narayan, 2021). Comprehensive quantification of multiple organelles in whole cells using DL segmentation has been reported for serial cross-sections of the mouse liver (Jiang et al., 2021) and for cultured cells (Heinrich et al., 2021), which suggests future possibilities for cell biology that may arise from intracellular morphomics. Thus, these AI approaches accelerate the acquisition of imaging data for further quantitative analysis.

### DL applications to medical and functional images

The use of DL in pathology has also advanced remarkably. Using AI to analyse tissue sections is often referred to as computational pathology (Laak et al., 2021). It has been reported that AI-based approaches can improve the diagnostic precision of various cancer types (Bera et al., 2019). For kidney disease assessments, the relationship between renal histology and the prognosis/severity of renal diseases has been examined using image recognition and comprehensive segmentation of the constituent tissues of renal samples, including human kidney biopsies (Hermsen et al., 2019; Bouteldja et al., 2021).

Intriguingly, recent DL results have been associated with biological functions such as the prediction of gene expression patterns and genetic mutations from histological images (Echle et al., 2021). The prediction of genetic mutation patterns in cancers has been achieved using morphology image recognition, which enables severity classification (Brück et al., 2021). Digital imaging studies have been conducted to explore the relationship between histology and gene expression patterns. Ash et al. (2021) used convolutional autoencoders and sparse canonical correlation analysis on paired histological images and bulk gene expression data and predicted genetic variation sets associated with tissue morphology. Schmauch et al. (2020) reported that HE2RNA, a model based on the integration of multiple data modes, can systematically predict RNA-seq profiles of tumours from whole-slide histological images alone. Howard et al. (2021) showed that DL-based features vary substantially across tissue submitting sites in the Cancer Genome Atlas for patients with six cancer subtypes. These multifactorial site-specific signatures may then lead to overoptimistic estimates of model performance. Diao et al. (2021) presented an approach for predicting clinically relevant molecular phenotypes from whole-slide images using human-interpretable image features and demonstrated prediction results for several molecular phenotypes, including the expression of multiple immune checkpoint proteins and homologous recombination deficiency. Future developments may lead to the prediction of gene expression at the nano-scale level. In addition, post-DL analysis should be used to determine which biologically meaningful information can be extracted from the quantification of morphological features.

## The blueprint for morphomics

The development of EM has placed nano-scale imaging data at the centre of morphological analysis. Large 2D and 3D datasets can be acquired with a millimetre-wide range at a nanometre resolution. However, previous morphological studies involving big data analysis have been limited to the analysis of individual datasets, sometimes of a single dataset of normal tissue, without any comparison. Although comparisons of several morphological features have been conducted in simple datasets, comparisons across datasets or among unbiased structural features are currently challenging.

In the morphome, as an omics field, creating a ‘reference morphology’ or a ‘reference EM dataset’ is needed, just as a reference genome is necessary at an early stage in the development of genomics studies. In this context, the reference must be statistically valid inside the 3D structures of tissues, cells, organelles, and their populations in the entire morphome. Indeed, the power of morphomics is demonstrated when appropriate reliable quantities of morphome are achieved without sampling biases. The construction of multiple 3D structures from tissues and cells by morphomics improves the statistical reliability of the morphome data. It is also necessary to observe the entire organisation macroscopically through a correlative methodology. Further, a standardized workflow including sample preparation, microscopic settings, and image analysis is established, and it will be possible to conduct larger-scale comparative studies that could have major biological implications. The use of bioinformatics methods and imaging databases will accelerate this process. In addition, further multi-omics analysis techniques that can bridge the gap between morphomics and other omics will be powerful tools. In particular, we expect the development of revolutionary methodologies that combine large-scale EM data analysis techniques with the analysis of genomic data such as gene mutation and expression data. According to recent reports, morphological information encompasses genomic profiles, and morphological information can also be predicted from genomic profiles, which implies that the morphomic data can be utilized across different hierarchies of omics. In addition to these image analyses utilizing DL, more recent advancements have led to the emergence of DL-based Large Language Models (LLM) (OpenAI, 2023), and the integration of various DL approaches is becoming an increasingly powerful tool. The progression of LLM is anticipated to enhance the interpretation of large-scale biological images and the application of diagnostic techniques derived from morphomics analyses.

By incorporating these new methodologies, the field of pathology is expected to progress rapidly, possibly through the identification of previously unknown structures, the quantification of rare events, the reclassification of diseases, and the automatic diagnosis of diseases. Furthermore, the amount of data that can be analysed is expected to increase dramatically with the development of automatic AI analysis.

## Supplementary Material

mjad081_Supplemental_Files
