# Supplementary material for: Morphomics via next-generation electron microscopy
Source: J Mol Cell Biol. 2023 Dec 26;15(12):mjad081. doi: 10.1093/jmcb/mjad081 (PMC11167312; doi:10.1093/jmcb/mjad081)
Supplement: mjad081_Supplemental_Files [file mjad081_supplemental_files.zip › Supplementary_Figures.pptx]

## Slide 1
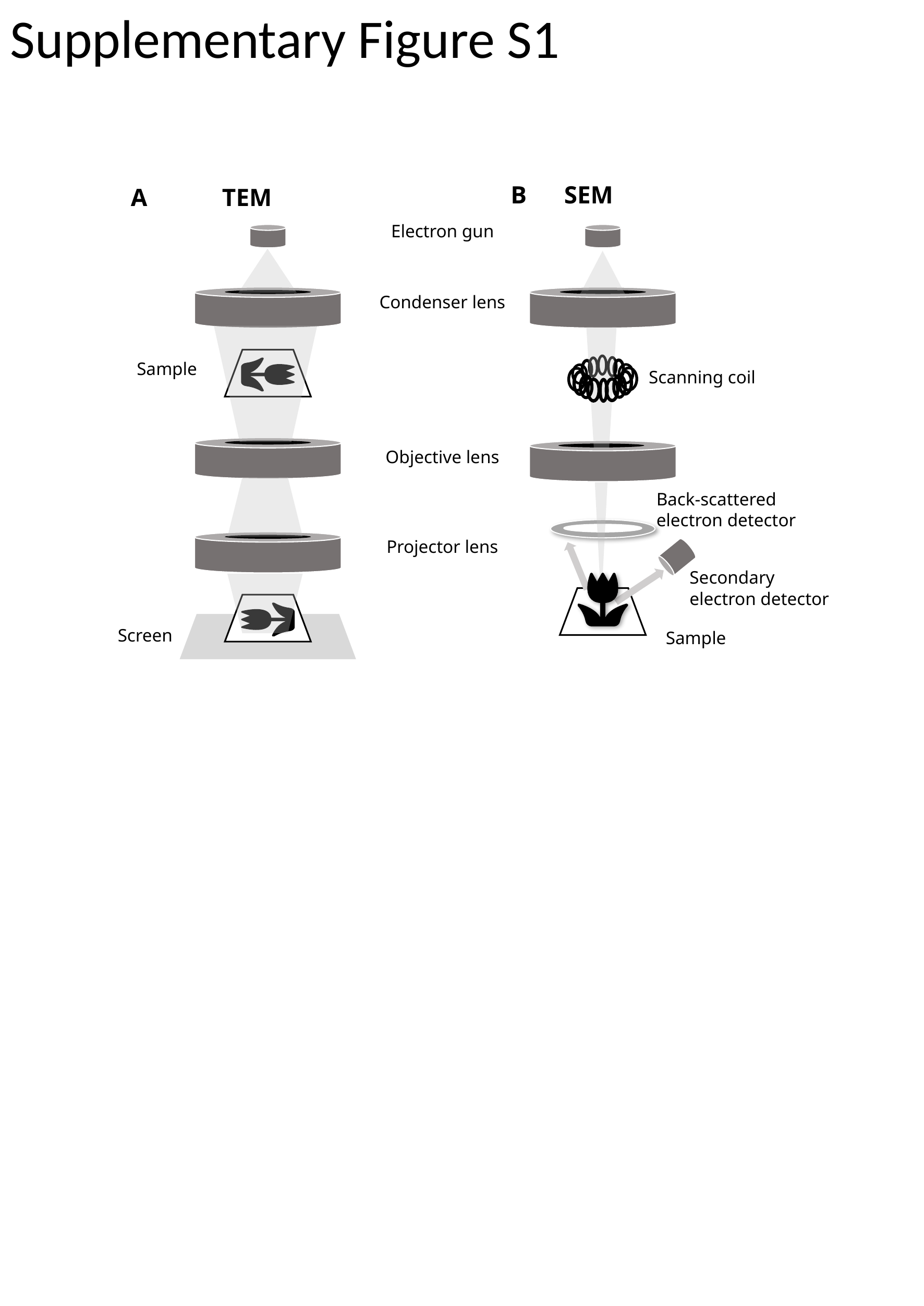

Supplementary Figure S1
B SEM
A TEM
Electron gun
Condenser lens
Sample
Scanning coil
Objective lens
Back-scattered
electron detector
Projector lens
Secondary
electron detector
Screen
Sample

## Slide 2
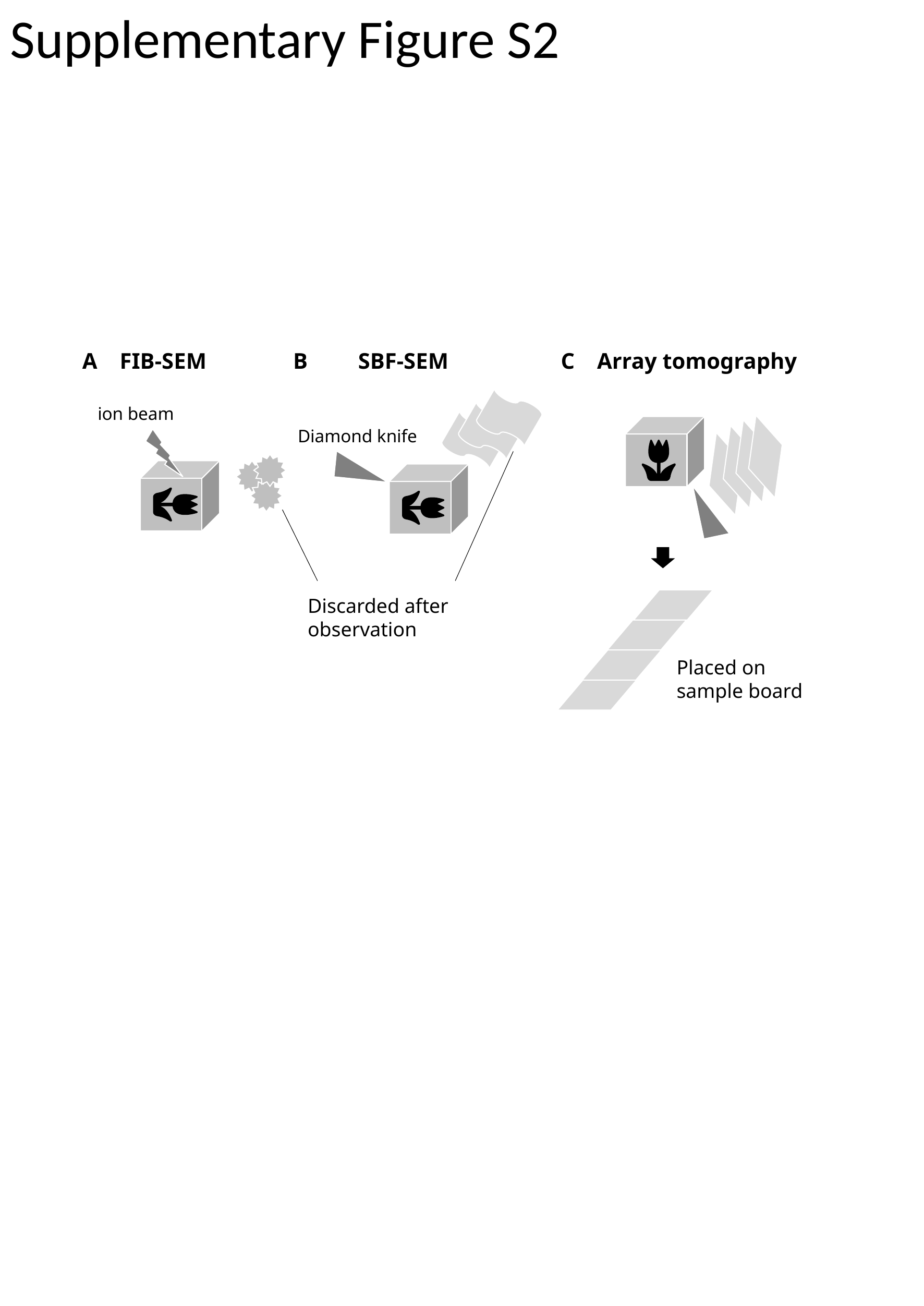

Supplementary Figure S2
A FIB-SEM
B SBF-SEM
C Array tomography
ion beam
Diamond knife
Discarded after observation
Placed on
sample board
